# Colorimetric aptasensor coupled with a deep-learning-powered smartphone app for programmed death ligand-1 expressing extracellular vesicles

**DOI:** 10.3389/fimmu.2024.1479403

**Published:** 2025-01-23

**Authors:** Adeel Khan, Haroon Khan, Nongyue He, Zhiyang Li, Heba Khalil Alyahya, Yousef A. Bin Jardan

**Affiliations:** ^1^ State Key Laboratory of Bioelectronics, School of Biological Science and Medical Engineering, Southeast University, Nanjing, China; ^2^ Department of Biotechnology, University of Science and Technology, Bannu, Pakistan; ^3^ Neuroscience and Neuroengineering Research Center, Med-X Research Institute, School of Biomedical Engineering, Shanghai Jiao Tong University, Shanghai, China; ^4^ Department of Clinical Laboratory, The Affiliated Drum Tower Hospital of Nanjing University Medical School, Nanjing, China; ^5^ Department of Exercise Physiology, College of Sport Science and Physical Activity, King Saud University, Riyadh, Saudi Arabia; ^6^ Department of Pharmaceutics, College of Pharmacy, King Saud University, Riyadh, Saudi Arabia

**Keywords:** extracellular vesicles, exosomes, programmed death ligand-1, PD-L1, lung cancer, AI -diagnostic, smartphone app

## Abstract

Lung cancer is a devastating public health threat and a leading cause of cancer-related deaths. Therefore, it is imperative to develop sophisticated techniques for the non-invasive detection of lung cancer. Extracellular vesicles expressing programmed death ligand-1 (PD-L1) markers (PD-L1@EVs) in the blood are reported to be indicative of lung cancer and response to immunotherapy. Our approach is the development of a colorimetric aptasensor by combining the rapid capturing efficiency of (Fe_3_O_4_)-SiO_2_-TiO_2_ for EV isolation with PD-L1 aptamer-triggered enzyme-linked hybridization chain reaction (HCR) for signal amplification. The numerous HRPs catalyze their substrate dopamine (colorless) into polydopamine (blackish brown). Change in chromaticity directly correlates with the concentration of PD-L1@EVs in the sample. The colorimetric aptasensor was able to detect PD-L1@EVs at concentrations as low as 3.6×10^2^ EVs/mL with a wide linear range from 10^3^ to 10^10^ EVs/mL with high specificity and successfully detected lung cancer patients’ serum from healthy volunteers’ serum. To transform the qualitative colorimetric approach into a quantitative operation, we developed an intelligent convolutional neural network (CNN)-powered quantitative analyzer for chromaticity in the form of a smartphone app named ExoP, thereby achieving the intelligent analysis of chromaticity with minimal user intervention or additional hardware attachments for the sensitive and specific quantification of PD-L1@EVs. This combined approach offers a simple, sensitive, and specific tool for lung cancer detection using PD-L1@EVs. The addition of a CNN-powered smartphone app further eliminates the need for specialized equipment, making the colorimetric aptasensor more accessible for low-resource settings.

## 1 Introduction

Lung cancer globally reigns as the deadliest form of cancer, causing over a million deaths each year (1). The threat of lung cancer is exacerbated by limitations in diagnostic capabilities, resulting in the disease being mostly detected in the late stages and thus becoming resistant to treatment more frequently. Approximately 15% of cases are diagnosed early, while the rest are diagnosed during the locally advanced or metastatic stage (2). In this context, it is pertinent to find biomarkers that facilitate the early diagnosis of lung cancer. Extracellular vesicles (EVs) have attracted immense attention as next-generation candidate biomarkers for pathologies such as cancer (3).

EVs are nanoscale vesicles with a double membrane ranging in size from 50 to 1,000 nm. All major types of cells are the fountainhead of EVs. EVs are abundant and stable in the majority of body fluids, such as blood, saliva, cerebrospinal fluid, tears, and urine (4). EVs contain versatile cargoes of proteins, nucleic acids, and lipids. Cancer cells have been shown to produce more EVs than normal cells. EVs can be valuable indicators of cancer progression and can serve as potential liquid biopsy-based non-invasive diagnostic biomarkers. Extensive research recognizes the fact that EVs are offering a new frontier for cancer diagnosis and treatment (3). EVs exhibit specific surface markers associated with distinct cancer types. Additionally, their cargo reflects the originating cancer’s molecular characteristics. Therefore, EVs are easily accessible, non-invasive, and sustainable packets of diagnostic and therapeutic information; EVs are simply a nanosized array of diagnostic and therapeutic tools (5).

Some cancers, such as lung cancer, can develop mechanisms to escape the immune system. Cancer cells accomplish this by expressing membrane proteins such as programmed death ligand-1 (PD-L1) to inactivate immune cells such as CD8^+^ T cells. Similar to cellular PD-L1, PD-L1-positive EVs released by cancer cells can also promote tumor growth by blocking PD-1 on CD8^+^ immune cells. Recent research suggests that tumor-derived exosomes appear in the bloodstream even during the early stages of tumor development and can influence the microenvironment, potentially promoting metastasis (6). Intriguingly, exosomes from lung cancer patients frequently display high levels of PD-L1 on their surfaces. This finding raises the possibility that PD-L1-expressing EVs (PD-L1@EVs) could serve as a biomarkers for the detection of lung cancer (7). Moreover, quantification of PD-L1@EVs can be valuable for forecasting the efficacy of the immunotherapy (8).

Because of the enormous potential of PD-L1@EVs as biomarkers, various techniques have been employed for its detection. Techniques such as flow cytometry (9), ELISA (10), and Western blotting (11) have shown promising results but suffer from limitations such as long laborious operation and large sample volume requirement. Nanoparticle tracking analysis (NTA) technology can also provide information on EVs’ size and abundance; however, it lacks the ability to precisely pinpoint the presence of tumor-specific markers adorning the EVs’ surface (12). Recent times have witnessed the gradual adoption of advance techniques like surface plasmon resonance (SPR) (13), microfluidic sensors (14), and surface-enhanced Raman spectroscopy (SERS) (15) for EV detection. Despite their promise, these emerging approaches suffer from significant drawbacks. However, ELISA remains the dominant approach for quantifying exosomal PD-L1, despite its limitations, for instance, high sample consumption and insufficient detection sensitivity (16). PD-L1@EVs levels are often lower than those of other exosomal marker proteins, hindering their detection by ELISA. Researchers have explored alternative methods to address these issues, including SPR—this technique offers promising sensitivity, but further development is needed for wider adoption (13). Electrochemical, fluorescent, and other technologies hold potential, but their application to PD-L1@EVs quantification is still under investigation (17–20). A crucial challenge across most detection methods is the pre-isolation of EVs through another laborious or expensive tool before detection (21). Researchers have reported magnetic beads with TiO_2_ capable of rapidly capturing EVs directly from the serum through a TiO_2_-membrane-phosphate mechanism (19, 22–24), eliminating the need for expensive instruments or reagents for EV isolation. Some techniques utilize PD-L1 antibodies, aptamers, or specific peptides to isolate PD-L1@EVs. However, these methods may be susceptible to interference from soluble PD-L1 (s-PD-L1), compromising the accuracy of detection (10).

Aptamers are short oligonucleotides that stand out for their exceptional ability to bind specifically to non-nucleic acid targets, earning them the title of “chemical antibodies”. However, compared to antibodies, aptamers offer several advantages, including lower production costs, reduced risk of immune response (immunogenicity), exceptional stability, and faster synthesis (25). The field of biosensors is witnessing a surge in the use of aptamers for the detection of diverse analytes ranging from proteins to RNA molecules. Integration of signal amplification strategies such as hybridization chain reaction (HCR) into aptamer-based biosensors presents a promising solution for enhancing the sensitivity (26).

HCR was first introduced by Dirks and Pierce in 2004 (27). HCR utilizes strategically designed hairpin DNA strands to achieve sensitive target detection. The HCR design includes oligonucleotides such as two hairpins (H1 and H2) and an initiator. The mode of operation is that the H1 stem loop structure is opened by the initiator, resulting in hybridization to develop a sticky end of H1, which, in turn, hybridizes with H2 to generate another sticky end. The process continues until H1 and H2 are depleted or the process reaches its threshold. Thus, HCR generates long double-stranded DNA helices containing numerous repetitive units (28). Remarkably, a single initiator molecule can spark this chain reaction, significantly amplifying the signal for robust target identification. HCR offers several advantages that have propelled it to the forefront of practical applications. Unlike traditional methods, it operates under isothermal conditions, simplifying the process and minimizing the environmental impact (29).

Furthermore, the exceptional ability of a few initiators to trigger the formation of numerous hairpins positions HCR as a compelling alternative to other DNA-based amplification techniques like polymerase chain reaction (PCR), rolling circle amplification (RCA), and catalytic hairpin assembly (CHA). Unlike PCR, RCA, and CHA, which rely on enzymes and temperature fluctuations for signal amplification, HCR offers a distinct advantage owing to its isothermal and enzyme-free operation (30). Moreover, HCR serves as a probe amplification technique, amplifying reporter molecules instead of the target itself. This approach minimizes the risk of cross-contamination often encountered in methods such as PCR and RCA, where amplicon carryover can lead to false-positive results. In recent years, HCR has found extensive application in the development of diverse biosensors, encompassing various detection methods, for instance, colorimetry, fluorescence, and chemiluminescence (31).

Colorimetric approaches have been used extensively for the detection of a wide range of analytes and are hailed for their simplicity, cost-effectiveness, portability, and sensitivity (32). In colorimetry, the color change can be observed with the naked eye and is qualitative in nature; however, for further quantitative analysis of chromaticity, a benchtop instrument such as a spectrophotometer or custom-designed reader is needed. As in colorimetric approaches, the signal can be detected with the naked eye; therefore, advanced image analysis techniques can eliminate the need for a spectroscopy device, avoid subjectivity, and enable quantitative analysis that is not possible through the naked eye. To deal with this situation, deep learning under the machine learning branch of artificial intelligence offers an interesting avenue. Deep learning models, such as convolutional neural networks (CNNs), can automatically analyze images with high accuracy, extract data, recognize patterns, and be precise prediction models (33). With the widespread usage of smartphones across the globe and their rapidly advancing capabilities, they have also found entry into the field of biosensing. They can assist both in capturing images and in deep-learning-based image analysis in the form of applications (apps) to pave the way for advanced detection (34). Such an approach has been successfully incorporated in detection strategies for many biological molecules such as enzymes (35), cells (36), nucleic acids (37), antigen–antibodies (38), and microorganisms (39). Recent literature shows smartphone integration, mainly with colorimetric and fluorescence assays (40). Images are taken to assess the color change, mostly a custom-made smartphone app leveraging the power of specifically trained deep learning or machine learning algorithms that quantitate the chromaticity through image analysis, and the result of the testing is displayed on the interface of the smartphone app; thus, the smartphone acts as an intelligent quantitative analyzer. CNN can automatically perform robust image processing and colorimetric data analysis, eliminating the need for various image condition optimizations such as lighting and other complicated adjustments to a certain extent, greatly saving resources and manpower, thus useful for the development of an intelligent framework for chromaticity analysis with minimal user intervention or additional hardware attachments (41).

Herein, we designed a simple colorimetric aptasensor for the detection of EVs@PD-L1 by combining (Fe_3_O_4_)-SiO_2_-TiO_2_ mag-nanoparticles with PD-L1 aptamer-triggered HCR, resulting in the addition of numerous HRPs. HRP uses dopamine (colorless) as a substrate and converts it into polydopamine (blackish brown colored) in the presence of H_2_O_2_ (a 300-fold enhanced reaction in a short time) (42). The change in chromaticity corresponds to the quantity of PD-L1@EVs. For the transformation of the qualitative colorimetric approach into a quantitative operation, we developed an intelligent deep-learning-powered quantitative analyzer for chromaticity in the form of a smartphone app named ExoP, thereby achieving an intelligent analysis of chromaticity with minimal user intervention or additional hardware attachments for the sensitive and specific quantification of PD-L1@EVs.

## 2 Materials and methods

### 2.1 Reagents

The reagents used in various experiments in this research are detailed here. Bovine serum albumin (BSA) was obtained from Sangon Biotech (Shanghai) Co., Ltd. PD-L1 aptamer (H0) and HI and H2 (both simple and biotin labeled) sequences obtained from reference (43) were ordered from Sangon Biotech (Shanghai) Co., Ltd and are tabulated in **Supplementary Table S1**. Other auxiliary reagents such as HEPES buffer and SuperBlock blocking buffer (cat log no: 37581) were ordered from ThermoFisher Scientific, and Streptavidin-labeled horseradish peroxidase (HRP) was from Bioss Antibodies. Tetraethoxysilane (TEOS) and tetrabutyltitanate (TBOT) were purchased from Sinopharm Chemical Reagent Co., Ltd (China). Dopamine was purchased from Sigma-Aldrich. Tween-20 was acquired from Beyotime. Salmon sperm DNA was ordered from Invitrogen. For the preparation of various buffers used in this study, we used pure water obtained through the Milli-Q benchtop water purification system. All chemicals were of analytical grade and were used as received without further purification.

### 2.2 Cell lines for EV isolation

A549 (human lung adenocarcinoma cells), BEAS-2B (human lung epithelial cells), and H596 cells were purchased from Shanghai Cell Bank, Chinese Academy of Sciences. To culture the cells, Gibco’s Dulbecco’s modified Eagle’s medium (DMEM) was used. Fetal bovine serum (FBS) and exosome-depleted FBS were delivered by Gibco and System Bioscience, respectively. The culturing of the cells (A549, H596, and BEAS-2B) was achieved at 37°C in 5% CO_2_ and 95% air using DMEM containing 10% FBS (44). Once the growing cells covered almost 85% of the culture plate surfaces, we discarded the used media, washed the plates thrice with PBS, and added fresh media (DMEM) supplemented with 10% exosome-depleted FBS. After the 24- to 48-h incubation time, the media was poured into 50-mL conical tubes, stored at −20°C, and later subjected to ultracentrifugation to collect EVs (45).

Ultracentrifugation-based isolation of EVs from media involves spinning of culture media successively at 300 × g for 10 min, then 2,000 × g for 10 min, and 10,000 × g for 30 min (in Beckman Coulter, Allegra X-30R) at 4°C to mitigate unwanted components such as cells, dead cells, and cell debris, respectively. Following these steps, the clarified supernatant was filtered using a 0.2-µm syringe filter. This filtered solution was then ultracentrifuged (100,000 × *g* for 90 min) at 4°C using a Beckman Coulter Optima XPN-100 centrifuge. The supernatant was removed, while the remaining pellet, enriched in EVs, was resuspended in 1 mL of PBS and subjected to another round of ultracentrifugation (100,000 × *g* for 90 min) at 4°C using a TYPE 90 Ti Rotor. Finally, the supernatant was removed and the precious EV pellet was resuspended in 100 µL of sterile-filtered (0.22 µm filter) PBS before being stored at −20°C for experiments to be conducted immediately and at −80°C for experiments to be performed later.

### 2.3 Production of (Fe_3_O_4_)-SiO2-TiO_2_ mag-nanoparticles

The (Fe_3_O_4)_-SiO_2_-TiO_2_ mag-nanoparticles were prepared following a combination of previously published protocols (19, 46). We prepared (Fe_3_O_4_)-SiO_2_-TiO_2_ mag-nanoparticles using Li et al.’s (24) protocol, including a classic recipe: the Stöber method. First, we took 0.3 g of Fe_3_O_4_ and mixed it up with 0.5 L of ethanol, 0.15 L of deionized water, and 0.01 L of ammonia. It was exposed to sonication for 15 min. Then, we added 4 mL of TEOS and continued to stir (1,500 rpm) the whole concoction for 10 h at 25°C.

Finally, (Fe_3_O_4_)-SiO_2_-TiO_2_ was made using a kinetics-controlled coating process. We started by taking 0.05 g of our (Fe_3_O_4_)-SiO_2_ and mixed it with an amalgam of 0.1 L of ethanol and 0.3 mL of ammonia (28%). Similar to before, the mixture was sonicated for 15 min. Then, we added 0.75 mL of TBOT in ethanol while constantly stirring (2000 rpm, 24 h, 45°C) the mix. Finally, the (Fe_3_O_4_)-SiO_2_-TiO_2_ mag-nanoparticles were washed 3× with water and ethanol and then dried in an oven overnight at 70°C, making them ready for utilization in future experiments.

### 2.4 Profiling and concentration of EVs

EVs’ shape and structure (morphology) were confirmed using transmission electron microscopy (TEM). Approximately 20 μL of the EV sample was carefully placed onto a copper grid. Next, we employed filter paper to gently remove any excess liquid. To enhance the contrast of the EVs in the image, a 2% phosphotungstic acid solution was dropped (5 min). Following that was a washing step using double-distilled water (5×) and then the copper grid was allowed to dry completely. Finally, the EVs were visualized using a high-powered JEM-2200CX TEM microscope from JEOL. For WB, protein concentration was assessed using a Qubit fluorometer from Invitrogen. Next, the EVs were subjected to a lysing step, by heating them in loading buffer at 95°C for 5 min. This process essentially breaks down the EVs and releases their protein cargo. The lysed proteins were then loaded onto a specialized gel (10% SDS-PAGE gel ordered from Sangon Biotech) specifically designed to separate proteins based on their size.

To capture these separated proteins, a polyvinylidene difluoride (PVDF) membrane, also ordered from Sangon Biotech, was employed to faithfully transfer the protein bands from the gel during a subsequent step. Subsequently, the membrane was blocked using a 2% non-protein blocking solution for 2 h. After blocking, the membrane was washed and dunked into a mixture of primary antibodies [CD9, TSG101 (ab275018 by Abcam), and PD-L1 by Proteintech] and kept at 4°C for 12 h. Excessive primary antibodies were washed (3×) away and the membrane was dunked in the solution of appropriate secondary antibodies (goat anti-rabbit, Abcam) at room temperature for 2 h.

Finally, the bands were visualized using the enhanced chemiluminescence (ECL) kit ordered from Sangon Biotech. To determine the EVs’ concentration and size distribution, we used an NTA machine (ZetaView, Particle Metrix, Diessen, Germany). EV samples were serially diluted (typically 1:100 to 1:1,000) using filtered PBS to ensure that the EV samples fell within the detectable range of the NTA machine. Subsequently, the ZetaView^®^ software automatically analyzed all the captured frames, ensuring that the outliers discarded the most relevant parameters; that is, the concentration of EVs and their average size were calculated.

### 2.5 Confocal microscopy and zetapotential

Performed via Leica Microsystems confocal microscope equipped with a 100× oil immersion objective, EVs were dyed using PKH26 dye following the manufacturer’s instructions. PBS was used as the control. A Zetasizer Nano series (Nano-ZS) instrument from Malvern was engaged to evaluate the surface charge of (Fe_3_O_4_)-SiO_2_-TiO_2_ mag-nanoparticles (0.5 mg), A549 cell-released EVs (10^8^ EVs/mL), and the complex formed between the mag-nanoparticles and EVs (0.5 mg/mL).

### 2.6 Magneto-capturing of EVs

EVs enriched from the A549 cell culture media were combined with (Fe_3_O_4_)-SiO_2_-TiO_2_ mag-nanoparticles for testing their EVs’ capturing strength, using a previously reported method based on the PKH26 dye (19). According to the method, we made two samples with the same number of EVs isolated from A549 cells. In both samples, EVs were dyed with PKH26 dye following the manufacturer’s instructions. A specific concentration of EVs (10^8^ EVs/mL) was used for this step. The fluorescence of these dyed EVs was then measured using a spectrophotometer. Next, the dyed EVs were mixed with (Fe_3_O_4_)-SiO_2_-TiO_2_ mag-nanoparticles. After separating the mag-nanoparticles and the attached EVs using a magnet, the fluorescence of the solution (supernatant) was measured. For accuracy, the experiment was repeated three times.

Now, to test that how well the (Fe_3_O_4_)-SiO_2_-TiO_2_ captured the EVs, we calculated the capture efficiency (%) using the following formula: capture efficiency (%) = (F2/F1) × 100. Here, F1 represents the initial fluorescence of the EVs solution before being enriched with the (Fe_3_O_4_)-SiO_2_-TiO_2_ mag-nanoparticles, and F2 represents the fluorescence after enrichment. The experiment was further expanded to determine the ideal concentration of (Fe_3_O_4_)-SiO_2_-TiO_2_ mag-nanoparticles and the optimal incubation time needed to achieve the best capture efficiency.

### 2.7 Gel electrophoresis analysis

To visualize the HCR product, we performed agarose gel electrophoresis and PAGE (Vazyme kit). For agarose gel electrophoresis, 2% agarose was used. H1 and H2 were separately pre-heated (at 95°C) for a short time and then left to cool at room temperature for an hour. For HCR, H0 (0.6 µM), H1 (2 µM), and H2 (2 µM) were mixed. Ten microliters was taken from each sample mixed with 2 µL of loading buffer. To load onto the gel, we took 10 µL from each sample and performed electrophoresis. Likewise for PAGE, 10 µL was taken from each sample and 2 µL of ×6 loading buffer was mixed. Later, 10 µL of the mixture was loaded into a 12% PAGE. Running was done at 100 V for 2 h in ×1 TBE buffer. After that, the gel was stained for 10 min in 50 mL ×1 TBE buffer with 5 μL of ×10,000 Gel-Red nucleic acid gel stain. The gels were then photographed using a Tanon 2500R gel imaging system.

### 2.8 Clinical testing

We obtained ethical approval and written consent from the participants to use their serum samples, which were collected at the clinical laboratory of the Drum Tower Hospital affiliated with Nanjing Medical University. A total of 42 samples were acquired from 21 healthy and 21 lung cancer patient samples; their clinical details are tabulated in **Supplementary Table S1** (**Supplementary Data Sheet 1**). Initially, a 0.22-µm filter attached to a syringe was used to filter the serum. Then, approximately 50 µL of the unaltered serum was incubated with (Fe_3_O_4_)-SiO_2_-TiO_2_ for 10 min to capture EVs. Subsequently, the remaining steps of the designed colorimetric aptasensor were performed. We used PBS as a blank control for comparison.

For comparison purposes, we followed the conventional plate ELISA protocol of Chen et al. (10) for PD-L1@EVs, with slight modifications. PD-L1 antibody (5 µg/mL) was coated to the wells of the plate and kept at 4°C overnight. The SuperBlock solution was used for blocking for 2 h. EVs isolated from clinical samples were added to the wells and incubated overnight. The detection antibody was added at a concentration of 1 μg/mL per well and incubated for 1 h at room temperature. Afterward, 100 μL of HRP-streptavidin, diluted 1:10,000 in SuperBlock blocking buffer, was added to each well and incubated for 15 min at room temperature before measurement.

### 2.9 Deep learning model for results analysis

Deep learning is preferred for complex tasks as it automatically extracts information from raw data to learn and train and then utilizes those data to make decisions about new unexposed data (47). Therefore, a deep learning model was established for chromaticity analysis. We acquired images of tubes from PD-L1@ EVs concentrations ranging from 0, 10^3^, 10^4^, 10^5^, 10^6^, 10^7^, 10^8^, 10^9^, and 10^10^ EVs/mL. The images were obtained through three different types of smartphones (iPhone X, Huawei mate 30 pro and Redmi Note 12 Turbo) under standard lab lights using different shooting angels and default camera settings from 40 cm height. However, the background was consistently kept white. Moreover, through data augmentation, we increased the images to 3,000 for better training of the deep learning model using various conditions such as color temperature, brightness modulation, contrast, pixel, image sharpening, Gaussian noise, and motion blur. Moreover, we consistently used 1.5 mL of Flex-Tube by Eppendorf throughout these experiments.

The deep learning model needs the optimization of the data format and parameters (48). It was trained using the TensorFlow and Keras frameworks (49, 50). The images were converted into NumPy arrays suitable for TensorFlow processing, and later converted from an RGB to an HSV format. Color thresholding was adjusted to define a broader hue range ([5, 40]) to capture light brownish to blackish colors. Saturation ([30, 255]) remains high to ensure a strong color presence. The value ([20, 255]) allows for variations in brightness within the color range. Focusing on the colored region (our sample) was achieved using the find_largest_contour function and the center of gravity. Based on the center of gravity, the images were first cropped to 600 × 600 pixels and then resized to 128 × 128 pixels. The pixels were normalized between 0 and 1 and saved as.pkl file. Then, we randomly divided the data between the training set (80%, 2,400 images) and the testing set (20%, 600 images).

CNN typically has convolution and pooling layers. The function of the convolution layer is to extract features from the images (51, 52). Then, it has a maxpooling layer to select the maximum value within a receptive field (53). After that, fully connected layers combine the features obtained by the previous two layers. Fully connected layers consist of a denser connection of neurons that are connected with neurons in the following layer (54). A CNN achieves nonlinearity by the application of an activation function. In general, CNN use rectified linear units (ReLU), but here we used a scaled exponential linear unit (SELU) activation function. SELU is a self-normalizing activation function. It is preferred for faster learning and prevention of gradient vanishing (55). The model configuration is tabulated in **Supplementary Table S2**.

### 2.10 Smartphone app (ExoP) development

The next step after training the model was the development of an app. For this purpose, we used our efficiently trained CNN model and TensorFlow Lite converter API to convert our Keras model into a TensorFlow Lite model (.tflite file), making it suitable for mobile phone devices. An app was developed using Android studio and Java. To integrate TensorFlow Lite, we used the TensorFlow Lite library for Android. For images obtained through the app to match the image size used for model development, we also preformed image preprocessing operation on the images imported through the app using the OpenCV_for_andriod library. Java was used to write images cropping from 600 × 600 to 128 × 128 pixels, as that of the image dimensions used for CNN training. BitmapFactory in Java programming language Android SDK (software development kit) was used to mitigate the impact on the image pixel quality. Finally, through the model’s prediction, the app’s user interface displays the estimated concentration of PD-L1@EVs.

## 3 Results

### 3.1 Design of the colorimetric aptasensor

**Figure 1:**
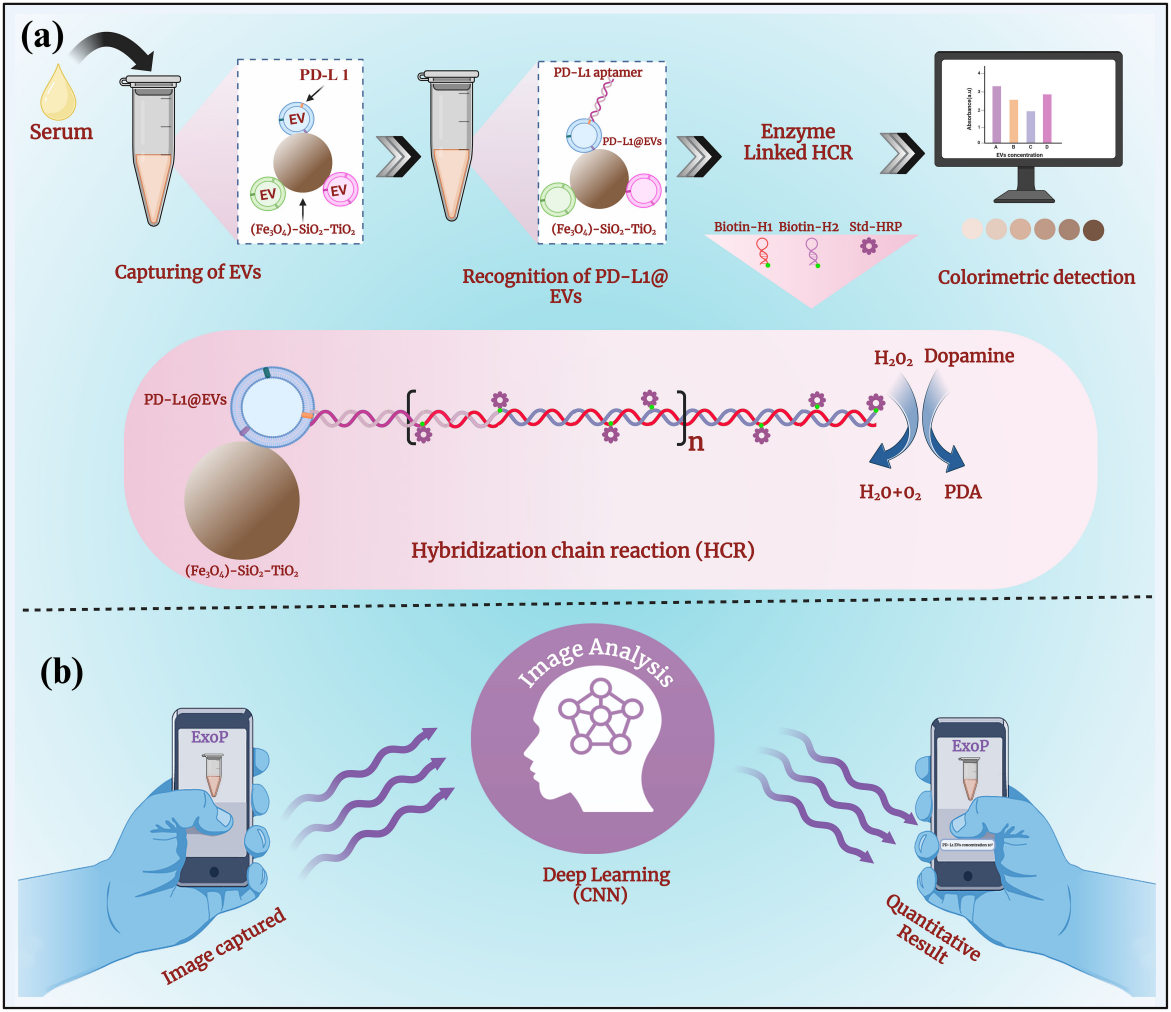
The schematic representation of a colorimetric aptasensor coupled with an intelligent deep-learning-powered smartphone App for quantitative analysis of chromaticity. **(A)** The aptasensor design combining the rapid magneto-capturing of EVs by (Fe_3_O_4_)-SiO_2_-TiO_2_ mag-nanoparticles. Aptamer recognition of PD-L1@EVs resulting in initiations of HCR with the addition of biotin-labeled H1 and H2 and streptavidin-labeled HRP for signal amplification through many HRPs. Change in chromaticity, as HRP converts dopamine to polydopamine in the presence of H_2_O_2_, corresponds to the concentration of PD-L1@EVs. **(B)** The deep learning (CNN)-powered smartphone app ExoP acts as a quantitative analyzer of chromaticity for predicting the concentration of PD-L1@EVs through image analysis.

The main design of the colorimetric aptasensor of EVs coupled with an intelligent deep-learning-powered smartphone app for quantitative analysis of chromaticity is depicted in **Figure 1**. As depicted in **Figure 1A**, EVs were magneto-captured by (Fe_3_O_4_)-SiO_2_-TiO_2_ mag-nanoparticles. After blocking with SuperBlock for 60 min, the PD-L1 aptamer that binds to EVs with the PD-L1 marker was added. Once the PD-L1 aptamer is attached, the addition of H1 and H2 (biotinylated) initiates the HCR reaction (PD-L1 apt + H1 + H2), forming a long self-assembled DNA concatemer. The numerous HRPs (streptavidin-conjugated) attached to the long strand then produce a colored signal upon the addition of the HRP substrate. HRP uses dopamine (colorless) as a substrate and converts it into polydopamine in the presence of H_2_O_2_ (a 300-fold enhanced reaction in a short time) (42). The change in chromaticity (absorbance at 400) corresponds to the quantity of PD-L1@EVs. To transform the qualitative colorimetric approach into a quantitative operation, we developed an intelligent deep-learning-powered quantitative analyzer for chromaticity in the form of a smartphone app named ExoP, thereby achieving the intelligent analysis of chromaticity with minimal user intervention or additional hardware attachments for the sensitive and specific quantification of PD-L1@EVs (**Figure 1B**). We envision that our smartphone app ExoP can fill in the gap between quantitative and qualitative measurements. Using a smartphone app as a portable quantitative analyzer of chromaticity is helpful to overcome the limitations of the colorimetric technique and improve quantitative analysis. From successful initial investigations, it can be postulated that the proposed methods hold promise for the rapid and accurate analysis of PD-L1-EVs in clinical serum samples, potentially paving the way for improved lung cancer detection.

### 3.2 Characterization and performance of Fe_3_O_4_-SiO_2_-TiO_2_ Mag-nanoparticles

The first important element of the colorimetric aptasensor is the development of (Fe_3_O_4_)-SiO_2_-TiO_2_ mag-nanoparticles. To analyze the morphology and composition of our (Fe_3_O_4_)-SiO_2_-TiO_2_ mag-nanoparticles, we employed the powerful duo of SEM and EDS. SEM generates a detailed image of the nanoparticle surface, revealing their size, shape, and overall morphology. EDS, on the other hand, reveals the elemental makeup of the nanoparticles, identifying the specific types of atoms that are present. **Supplementary Figure S1A** shows the images taken with SEM and revealed that the (Fe_3_O_4_)-SiO_2_-TiO_2_ nanoparticles were uniformly round with a bumpy surface made of many tiny clumps of TiO_2_. Furthermore, EDS (**Supplementary Figure S1B**) confirmed the formation of (Fe_3_O_4_)-SiO_2_-TiO_2_. The silica layer acted as a bridge, allowing for the later addition of TiO_2_.

The XRD report shown in **Supplementary Figure S1C** suggests that the particles are crystalline and have the anatase phase. Peaks at 2 theta 30.88, 35.22, 43.06, 53.5, 57.1, 62.72, and 74 correspond to the planes of the cubic phase of Fe_3_O_4_ and anatase phase of TiO_2_ (JCPDS No. 21-1272). The diffraction peak of (Fe_3_O_4_)-SiO_2_ showed no significant change compared with Fe_3_O_4_ because the coating layer SiO_2_ also belongs to an amorphous body. The XRD results suggest the successful formation of (Fe_3_O_4_)-SiO_2_-TiO_2_. FTIR-based examination of (Fe_3_O_4_)-SiO_2_-TiO_2_ nanoparticles (**Supplementary Figure S1D**) showed peaks at 802 and 1,068 cm^−1^, which confirm the presence of silica coating on the magnetic nanoparticles. After the addition of TiO_2_ to the surface, a new peak appeared in the FTIR spectrum at 970 cm^−1^. This new peak indicates vibrations between the titanium and oxygen atoms, proving the presence of the TiO_2_ layer. Peaks in the range of 500–700 cm^−1^ can be attributed to Ti–O–Ti and Ti–O–Si vibration.

### 3.3 Characterization of EVs with PD-L1

**Figure 2:**
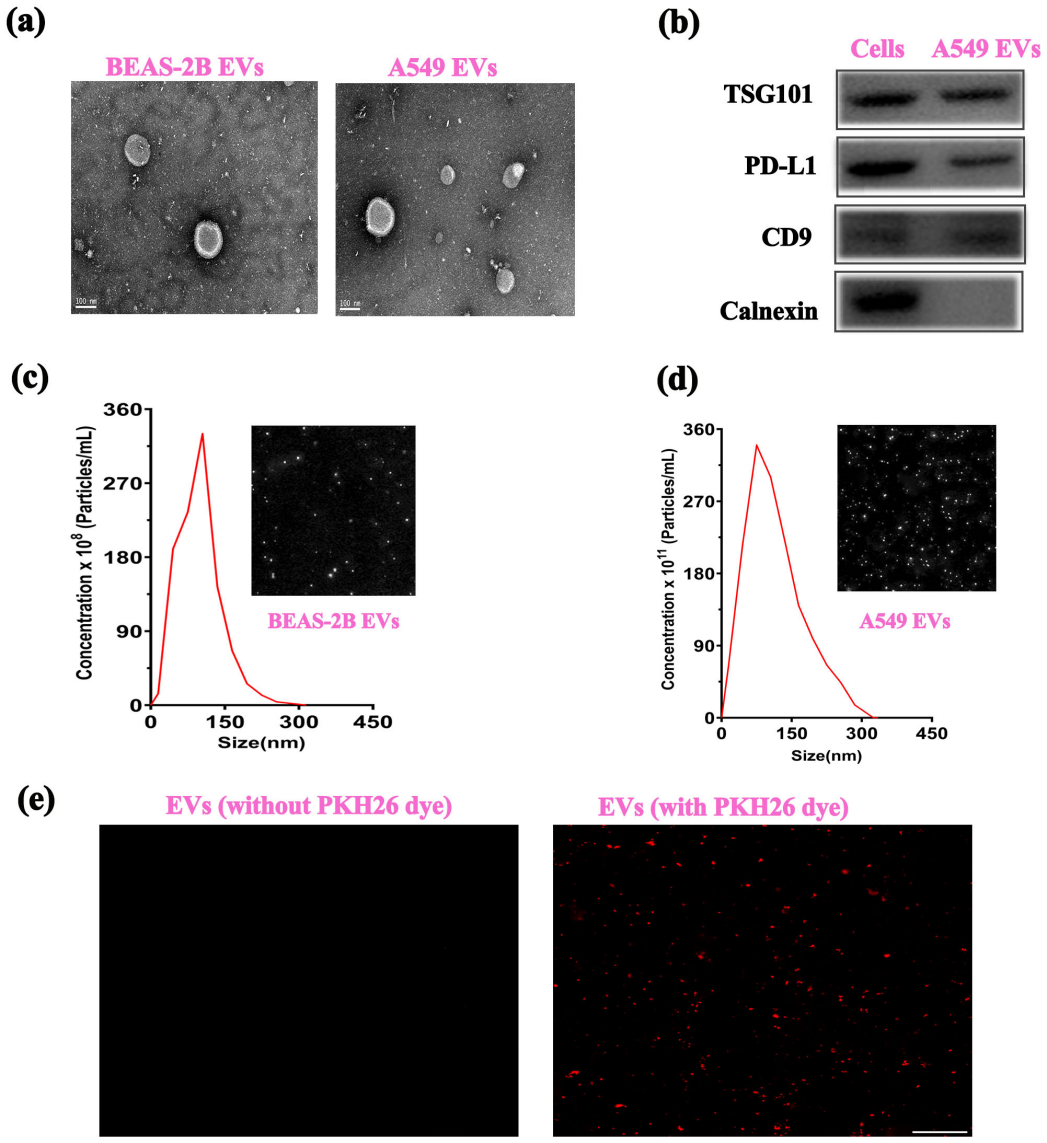
Characterization of extracellular vesicles. **(A)** TEM images of EV morphology, **(B)** Western blot confirmation of EV markers. NTA analysis of the concentration and size distribution of **(C)** BEAS-2B EVs and **(D)** N A549 EVs. **(E)** Membrane labeling of EVs using the PKH26 dye.

It has been recommended by MISEV 2023 guidelines to provide detailed profiling of EVs and their concentration. EVs acquired from A549 cells were assessed as cancer-specific EVs. While EVs obtained from the Beas-2B cells were assessed as normal control. TEM is one of the most widely used technologies for assessing the morphology of EVs. TEM is hailed for its prowess in detecting EVs, irrespective of size. The images obtained via TEM confirmed the presence of vesicular structures (**Figure 2A**). Another important aspect of EVs is to confirm the presence of EV-specific markers and cancer-specific markers. WB has been widely recommended for the detection of proteins in EV-containing preparations. It can be seen in **Figure 2B** that markers specific to EVs such as CD9 and TSG 101 are present. Likewise, the negative marker calnexin can also be observed to be present on cells but missing on EVs. We also observed the presence of PD-L1 markers both on lung cancer cell lysate (A549) and the EVs derived from those cells. NTA is an optical technique that provides information about EVs’ size and abundance. **Figures 2C, D** show the NTA analysis of the concentration and size of EVs obtained from BEAS-2B (10^8^ particles/mL) and A549 cells (10^11^ particles/mL), respectively. In **Figure 2E**, EVs can be seen visualized through confocal microscopy using a membrane binding specific dye, PKH26. From the above panels, it can be concluded that we obtained both BEAS-2B EVs and A549-derived EVs at high concentrations for conducting further experiments.

### 3.4 Rapid capturing of EVs by (Fe_3_O_4_)-SiO_2_-TiO_2_

**Figure 3:**
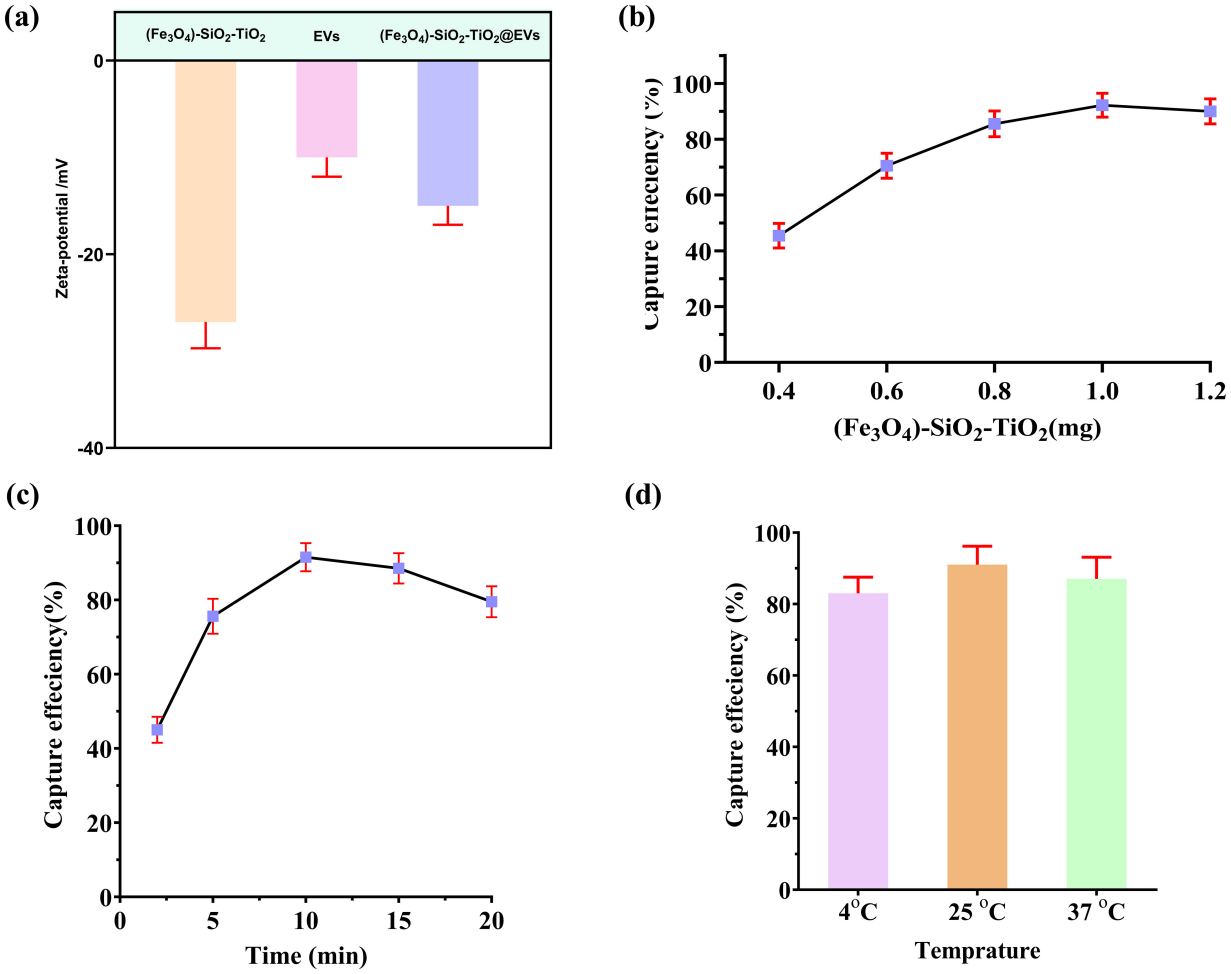
Magneto-capturing of EVs by (Fe_3_O_4_)-SiO_2_-TiO_2_. **(A)** Changes in the zetapotential of the (Fe_3_O_4_)-SiO_2_-TiO_2_ after binding EVs. **(B)** Selection of the ideal amount of (Fe_3_O_4_)-SiO_2_-TiO_2_ mag-nanoparticle for EV capturing. **(C)** Selection of ideal time duration for (Fe_3_O_4_)-SiO_2_-TiO_2_ mag-nanoparticles required for EV capturing. **(D)** Selection of optimum temperature required for (Fe_3_O_4_)-SiO_2_-TiO_2_ mag-nanoparticles to capture EVs.

One of the cornerstones of colorimetric aptasensor design is (Fe_3_O_4_)-SiO_2_-TiO_2_-mediated EV capture. Hence, we used the zetapotential techniques to confirm this. A significant change in the potential of mag-nanoparticles can be observed after the introduction of EVs, providing evidence of mag-nanoparticles binding the EVs (**Figure 3A**). Next, we systematically investigated the optimum quantity of (Fe_3_O_4_)-SiO_2_-TiO_2_ and the incubation time required for optimal capture efficiency. Varying concentrations of (Fe_3_O4)-SiO_2_-TiO_2_ (0.2–1.6 mg) were employed to determine the amount necessary for maximum capturing of PKH26-labeled EV. The fluorescence intensity was measured before and after the addition of (Fe_3_O_4_)-SiO_2_-TiO_2_. As depicted in **Figure 3B**, a dosage of 1 mg yielded an optimal capture efficiency exceeding 90%. Further increments in bead concentration resulted in negligible improvements.

Incubation time of the (Fe_3_O_4_)-SiO_2_-TiO_2_ for peak capture efficiency was optimized using PKH26-labeled EVs. Incubation durations ranging from 2 to 20 min were evaluated. The maximum capture efficiency was attained at 10 min, with negligible gains observed beyond this point (**Figure 3C**). In addition, the temperature for best capture efficiency of (Fe_3_O_4_)-SiO_2_-TiO_2_ was optimized using various temperature conditions (4°C, 26°C, and 37°C). The mag-nanoparticles exhibited the best capturing efficiency at 25°C (**Figure 3D**).

### 3.5 HCR optimization experiments

**Figure 4:**
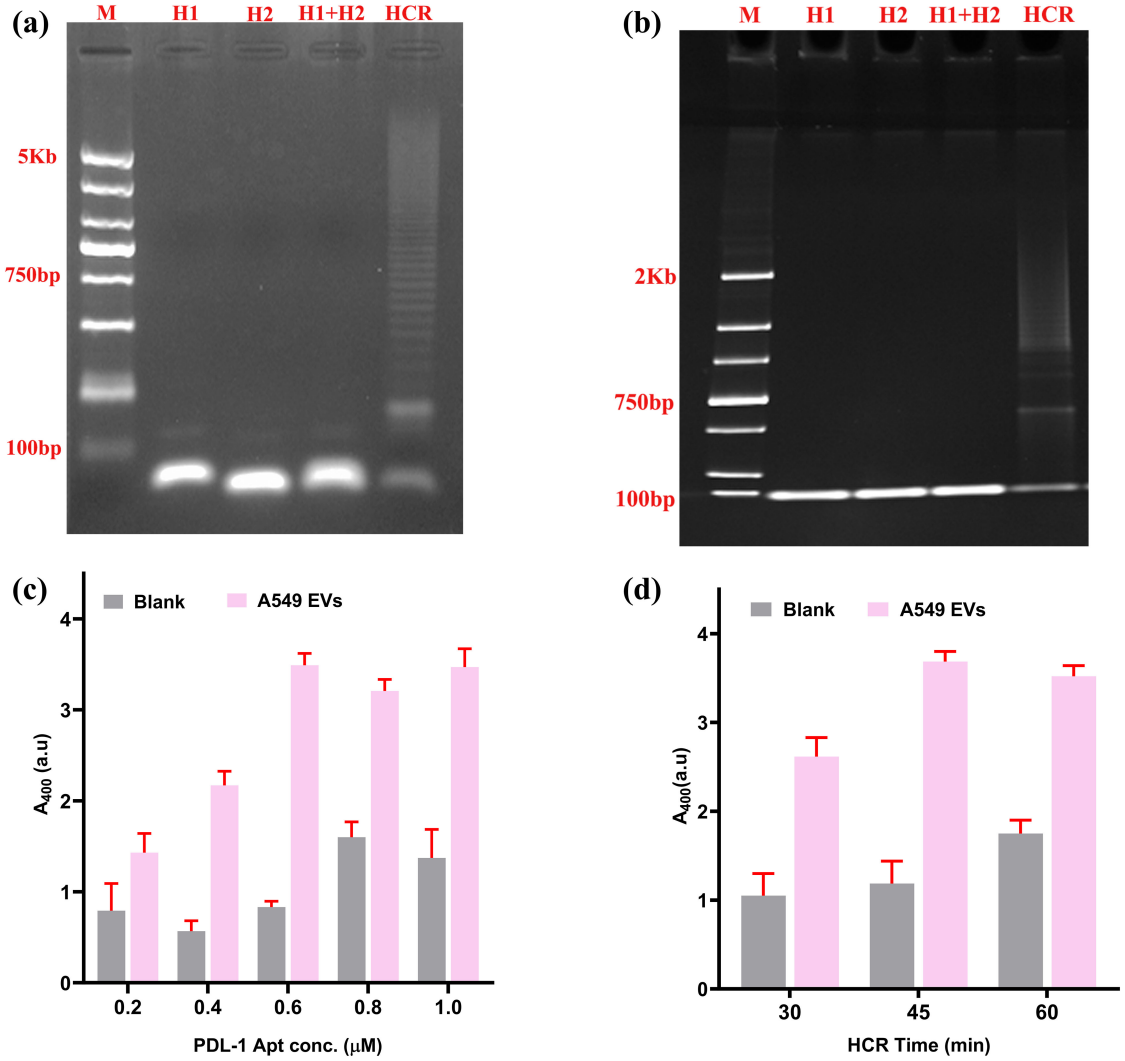
Selection of ideal concentration of aptamer and time for HCR. **(A)** Agarose gel confirmation for HCR reaction. **(B)** PAGE (polyacrylamide gel electrophoresis) confirmation of HCR. **(C)** Selection of ideal aptamer concentration of HCR. **(D)** Selection of ideal time duration required for HCR reaction.

To visually confirm the HCR, we performed Agarose gel electrophoresis (**Figure 4A**) and PAGE (**Figure 4B**). Both experiments provided visual proof of the HCR.

The PD-L1 aptamer (H0) has a central role for the successful initiation of HCR. A series of aptamer concentrations (H0 concentration from 0.2 to 1 µM) were evaluated against a constant concentration of EVs (10^9^ EVs/mL) as the experimental sample, with PBS acting as a blank control. The optimal signal-to-noise ratio was achieved at 0.6 µM concentration (**Figure 4C**). Subsequently, the optimal incubation time for the HCR reaction was optimized to maximize the S/N ratio. Three durations (30, 45, and 60 min) were tested. As illustrated in **Figure 4D**, the highest S/N ratio was attained at 45 min, with progressively higher background signals observed for longer HCR times.

### 3.6 Analytical performance of the colorimetric aptasensor for PD-L1@EVs

After optimization, we explored the sensor’s analytical parameters such as sensitivity, specificity, and accuracy for detecting PD-L1@EVs.

**Figure 5:**
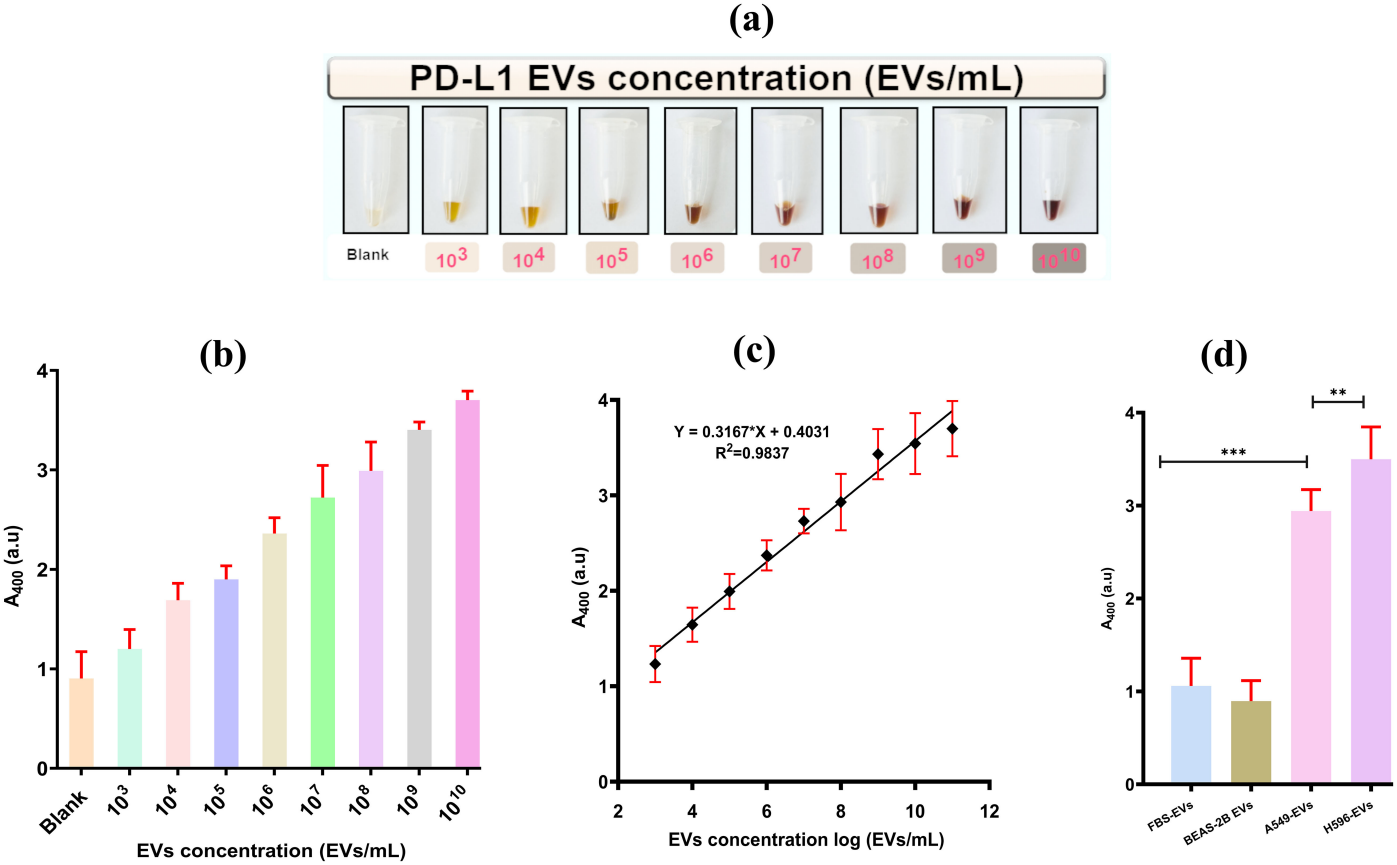
Visualization of colorimetric aptasensor analytical performance. **(A)** Change in chromaticity with the increase in the concentration of PD-L1@EVs. **(B)** Aptasensor analytical performance using various concentrations of A549 EVs (0–1010). **(C)** Sensitivity of the aptasensor and the linear relationship of chromaticity (absorbance at 400) with the increasing concentration of A549-EVs. **(D)** Graphical representation of aptasensor specificity (FBS, fetal bovine serum). **,*** P less than 0.05 and P less than or equal to 0.0001.

A broad range of A549 EVs concentrations (10^3^–10^10^ EVs/mL) were used for analysis. The peak absorbance at 400 nm, indicative of color intensity, was precisely calibrated using a spectrophotometer. A change in color and the corresponding absorbance (at 400 nm) were recorded for various concentrations of A549-EVs as well as for a blank sample containing PBS without EVs as in **Figures 5A, B**.

It can also be seen in **Figure 5C** that the change in color intensity/absorbance and the concentration of PD-L1@EVs are related in a linear fashion ranging from 10^3^ to 10^8^ EVs/mL following the equation *Y* = 0.3167*Log *N* + 0.4031, where *Y* stands for peak absorbance and *N* stands for the number of EVs. Similarly, the LOD was estimated by incorporating the typical three times the standard deviation of blank samples in the above equation. The LOD was estimated to be 3.6×10^2^ EVs/mL. It is of paramount importance for the sensor to be highly specific for target detection. We checked the sensor’s specificity by using EVs obtained from a variety of sources, such as EVs from the serum of a healthy volunteer (HS EVs), EVs from BEAS-2B cells (PD-L1-negative EVs), A549 cells, and H596 cells (two PD-L1-positive EVs). As can be seen in **Figure 5D**, high absorbance was recorded for the PD-L1@ EVs as compared to BEAS-2B EVs and FBS-derived EVs. Thus, it can be inferred that the designed colorimetric aptasensor demonstrated high specificity for the detection of PD-L1@EVs.

### 3.7 Colorimetric aptasensor performance with clinical samples

**Figure 6:**
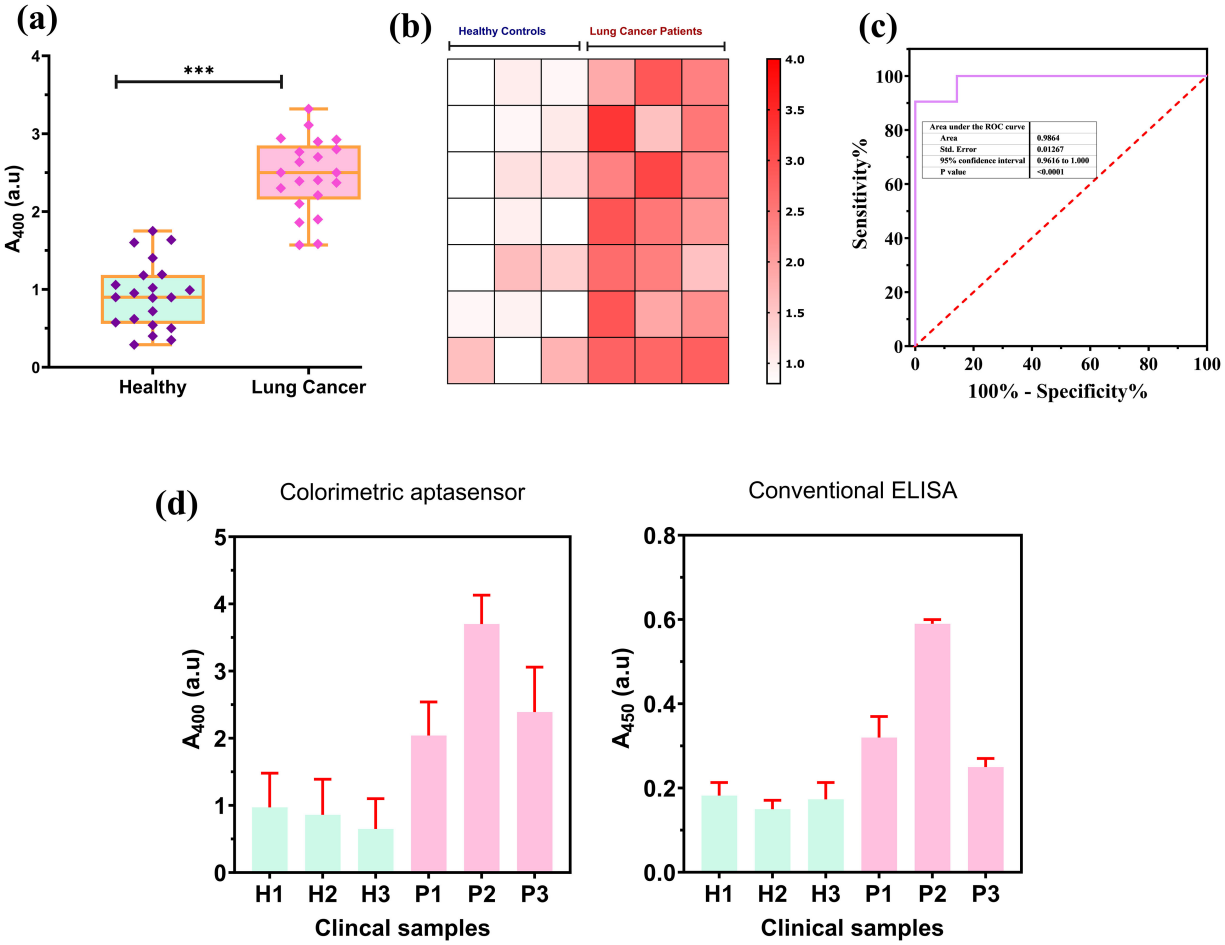
Colorimetric aptasensor performance on clinical samples. **(A)** A significant deviation exhibited in absorbance at 400 nm for healthy (n = 21) and lung cancer (*n* = 21) patients. **(B)** Heatmap-based visualization of absorbance difference between healthy and lung cancer patients. **(C)** ROC curve for the aptasensor performance on the clinical samples. **(D)** Colorimetric aptasensor performance comparison with conventional ELISA for PD-L1@EVs using clinical samples. *** P less than 0.05 and P less than or equal to 0.0001.

A critical aspect is to test the performance of the designed colorimetric aptasensor using complex clinical samples, such as serum. We tested the colorimetric aptasensor using a total of 42 clinical serum samples, of which 21 samples belonged to the healthy individuals and 21 belonged to the lung cancer patient group. Detailed clinical information is provided in **Supplementary Table S1** (Supplementary Materials). In **Figure 6A**, the absorbance values were significantly lower (*p*< 0.001, Mann–Whitney *U* test) for the serum samples belonging to the healthy group compared to the lung absorbance recorded for serum samples belonging to the lung cancer patients’ group. We also explained the differences between the absorbance values of serum of healthy and lung cancer patients through a heatmap. The heatmap in **Figure 6B** visually differentiated between healthy and lung cancer samples, with darker colors representing high concentrations of PD-L1@EVs. The ROC curve was also established to evaluate the performance of the aptasensor with the clinical samples as shown in **Figure 6C**. These findings corroborate the established notion that serum from lung cancer patients harbors the greatest abundance of PD-L1@EVs than healthy controls lacking detectable PD-L1@EVs concentration (56). To validate the performance of the newly designed aptasensor, we compared it with the conventional plate ELISA for PD-L1 proposed by Chen et al. (10), using six clinical serum samples, comprising three healthy and three lung cancer samples (**Figure 6D**). Both methods showed similar results in differentiating lung cancer serum samples from healthy samples, but our method takes a shorter time than the conventional plate ELISA and does not require a separate purification step.

### 3.8 Performance evaluation of the deep learning CNN model

**Figure 7:**
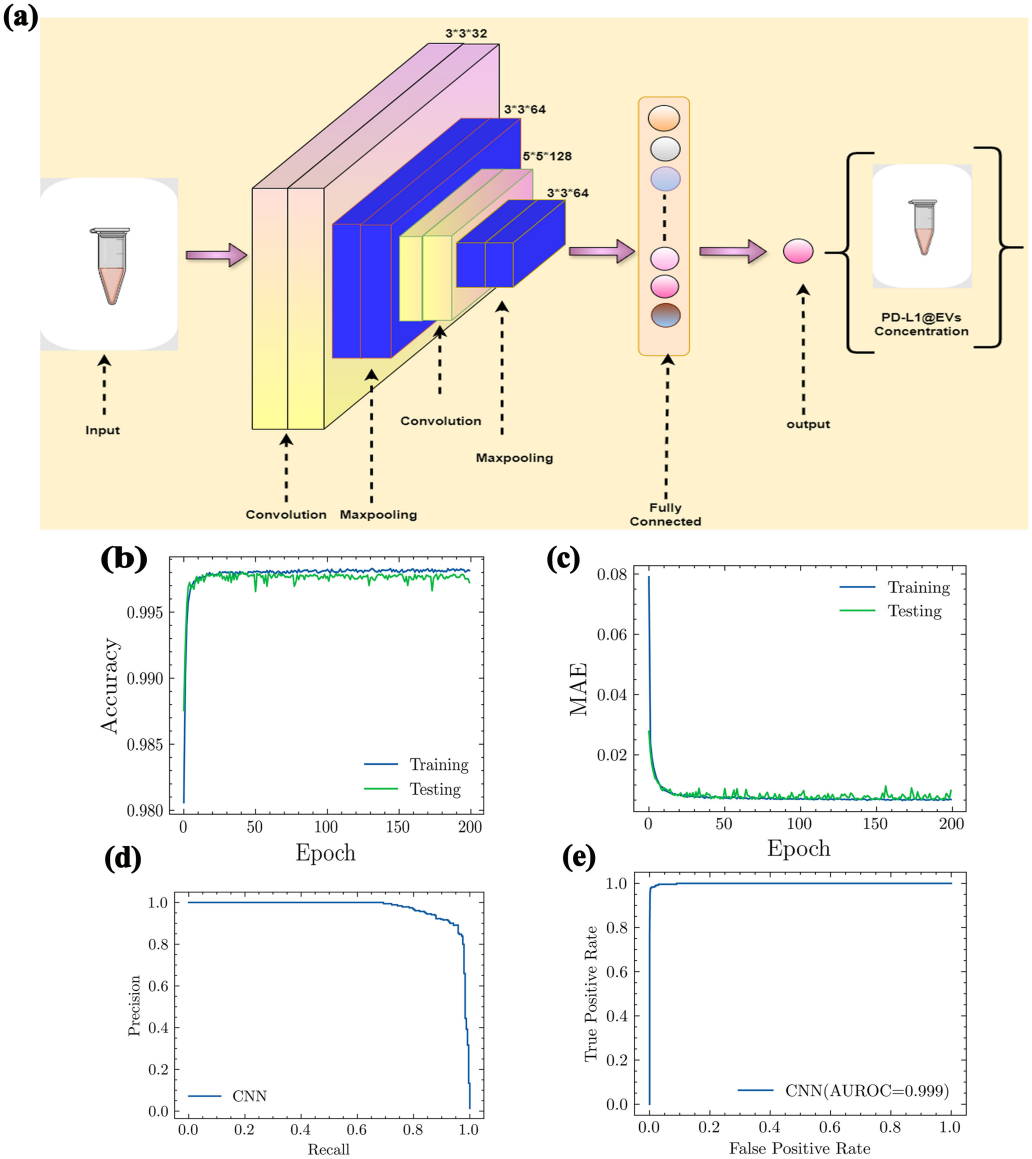
The architecture of CNN model and its performance evaluation. **(A)** Proposed CNN model architecture. **(B)** Accuracy, **(C)** MAE, **(D)** precision and recall curve, and **(E)** AUROC (AUROC, area under the receiving operating curve; MAE, mean absolute error).

The proposed model (the architecture is shown in **Figure 7A**) was checked for its ability to quantify the concentration of the PD-L1@EVs by analyzing the chromaticity of the tubes. We utilized a distinct confusion matrix derived from four matrices (1): true positives (TP), (2) true negatives (TN), (3) false negatives (FN), and (4) false positives (FP). TP indicates the correct identification of the actual concentration of PD-L1@EVs. TN indicates the correct identification of blank tubes without PD-L1@EVs. FN indicates incorrect prediction of PD-L1@EVs concentration as blank. FP indicates the incorrect prediction of the actual concentrations of PD-L1@EVs.

Using the values of the aforementioned four metrics, we further evaluated the following performance metrics for our deep learning model. A very straightforward metric for model evaluations is accuracy. It can be evaluated as


(1) $$\text{Accuracy}=\frac{\text{TP}+\text{TN}}{\text{TP}+\text{TN}+\text{FP}+\text{FN}}\;$$

In **Figure 7B**, it can be clearly seen that our deep learning model showed excellent accuracy (99%) in predicting the concentration of PD-L1@EVs by analyzing the chromaticity of the tube. Mean absolute error (MAE) measures the performance of the model as the average magnitude of the difference between predicted values and actual values. It can be calculated as:


(2) $$\text{MAE}=\frac{1}{m}\sum_{i-1}^{m}|\left(pi-yi)\right|$$

Here, *m* denotes the number of samples, *yi* denotes the true value, and *pi* denotes the predicted value. The MAE value shown in **Figure 7C** confirms the excellent capability of the model.

Another important graph is the precision–recall curve (PRC), which visually represents the effectiveness of a classification model at different levels of sensitivity. It assesses the relationship between two crucial aspects: precision and recall. Precision provides an assessment of the proportion of accurate positive predictions made by a model. In simpler terms, it gauges the accuracy of a model in predicting genuine positive cases. Recall, on the other hand, denotes the actual concentrations that have been accurately identified by the model (**Figure 7D**).

For a better understanding of model performance, we evaluated the F1 score. The F1 score is the harmonic mean of precision and recall. Precision, recall, and F1 score can be evaluated using the following equations, respectively:


(3) $$\text{Precision}=\frac{\text{TP}}{\text{TP}+\text{FN}}$$

(4) $$\text{Recall}=\frac{\text{TP}}{\text{TP}+\text{FN}}$$

(5) $$\text{F}1\text{ Score}=2\times \frac{\text{Precision}\times \text{Recall}}{\text{Precision}+\text{Recall}}$$

The area under the receiver operating characteristic curve (AUROCC) is a frequently used metric for assessing a model’s ability to discriminate across classes. A model with a higher AUROC is generally considered more useful and robust (**Figure 7E**).

**Figure 8:**
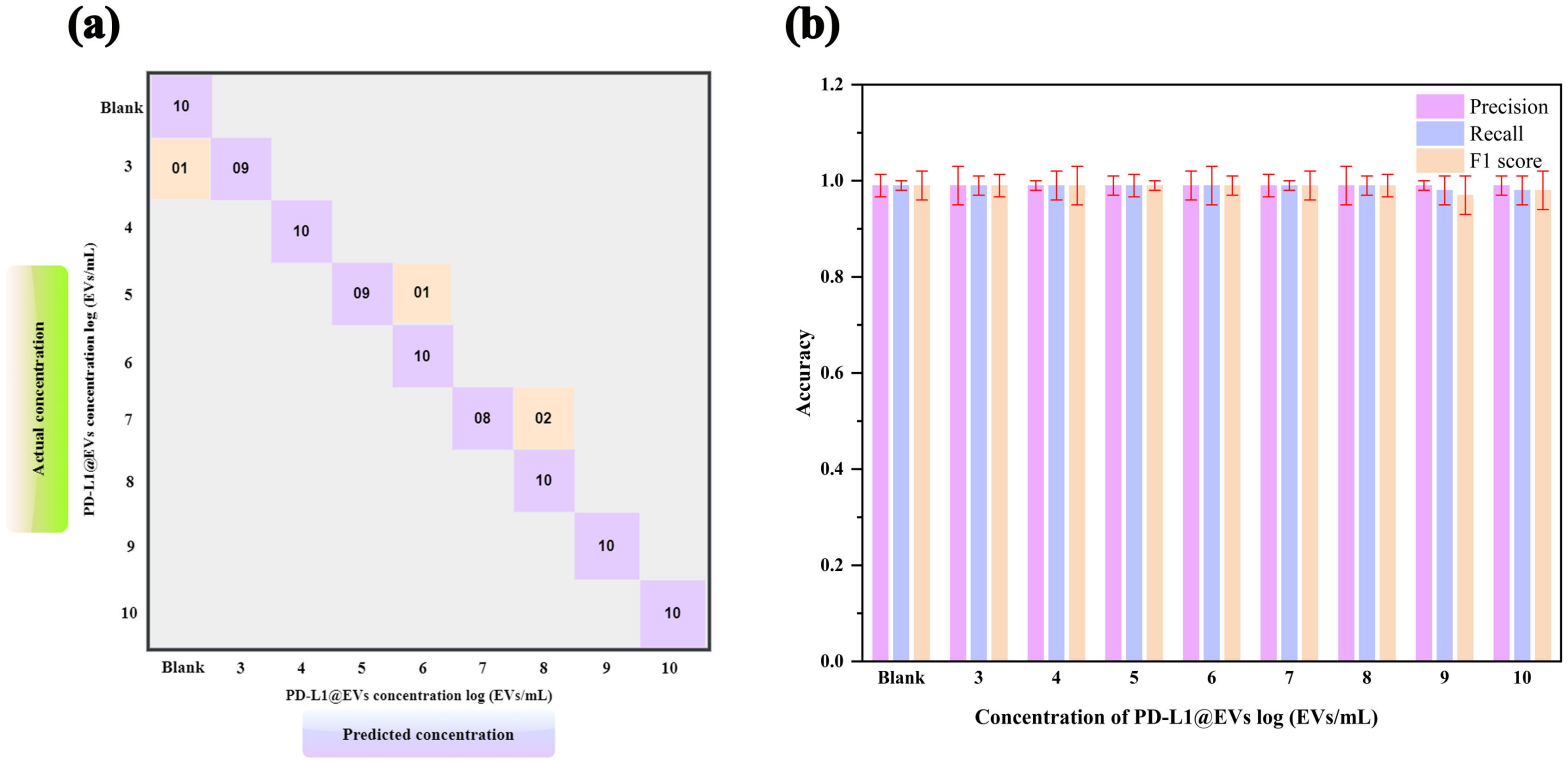
Model evaluation using 10 samples of various concentrations. Confusion matrix **(A, B)** precision, recall, and F1 score of the trained CNN model to performance analysis of chromaticity images for quantification of PD-L1@EVs.

To further validate our model’s ability to accurately identify the concentration of PD-L1@EVs, we used 10 images for each PD-L1@EVs concentration ranging from 0 to log 10 EVs/mL to find out the performance of the deep learning model (**Figure 8**).

As can be seen in the confusion matrix showing the relationship between the actual (true) concentration and predicted concentration of PD-L1@EVs (**Figure 8A**), 1 out of 10 tested samples at log 3 EVs/mL were misclassified as no concentration, 1 out of 10 tested samples at log 5 EVs/mL were misclassified as log 6 EVs/mL, and 2 out of 10 tested samples were misclassified as log 8 EVs/mL. The other concentration results are located diagonally in the confusion matrix, which means that they were correctly classified. **Figure 8B** depicts the model metrics for various concentrations of PDL1@EVs. From the exceptional metrics results, it can be inferred that the model functioned exceptionally well.

### 3.9 Performance evaluation of the ExoP smartphone app

**Figure 9:**
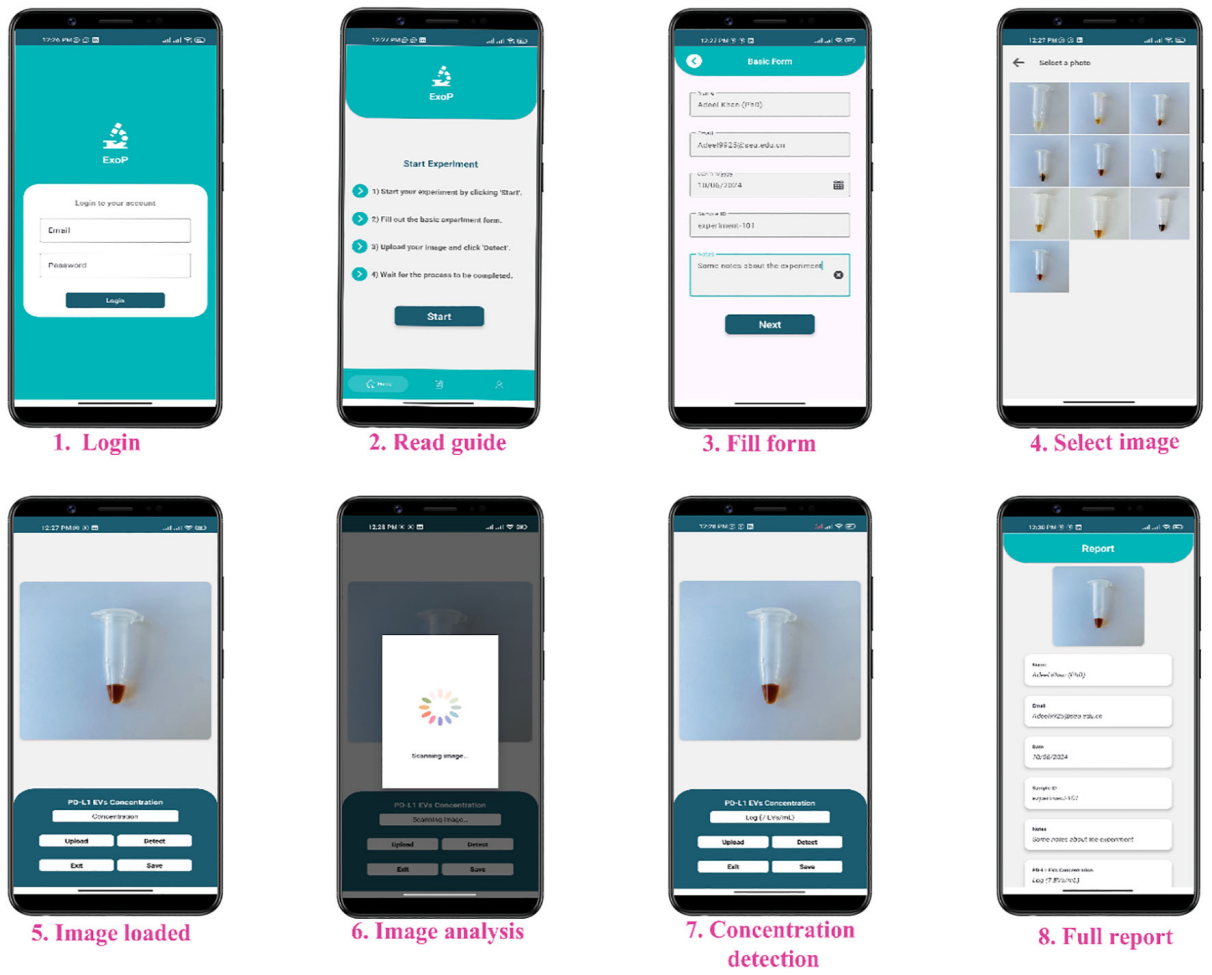
The user interface of the ExoP app.

To ensure consistent evaluation of our colorimetric aptasensor readouts, we also designed a smartphone app called ExoP utilizing a CNN that can robustly predict the concentration of PD-L1@EVs by analyzing the chromaticity of the tubes from the images. The ExoP user interface can be seen in **Figure 9**. This eliminates discrepancies associated with the colorimetric approach, such as lack of quantitative data without specialized equipment and inter-observer variability due to human subjectivity during visual interpretation (57). The key component behind the ExoP app is the CNN model, which has superior capabilities in gathering information from input data for learning and has been able to perform the task of distinguishing different concentrations of PD-L1@EVs from the tubes’ chromaticity on which it was not trained before. In essence, the ExoP app automates the quantitative interpretation of colorimetric aptasensor chromaticity for PD-L1@EVs concentration, making it more convenient and guaranteeing uniformity in the results. It is worth noting that the deep learning model can accurately analyze tube chromaticity, and the ExoP app can provide real-time quantitative values on the phone screen with high precision and accuracy.

**Figure 10:**
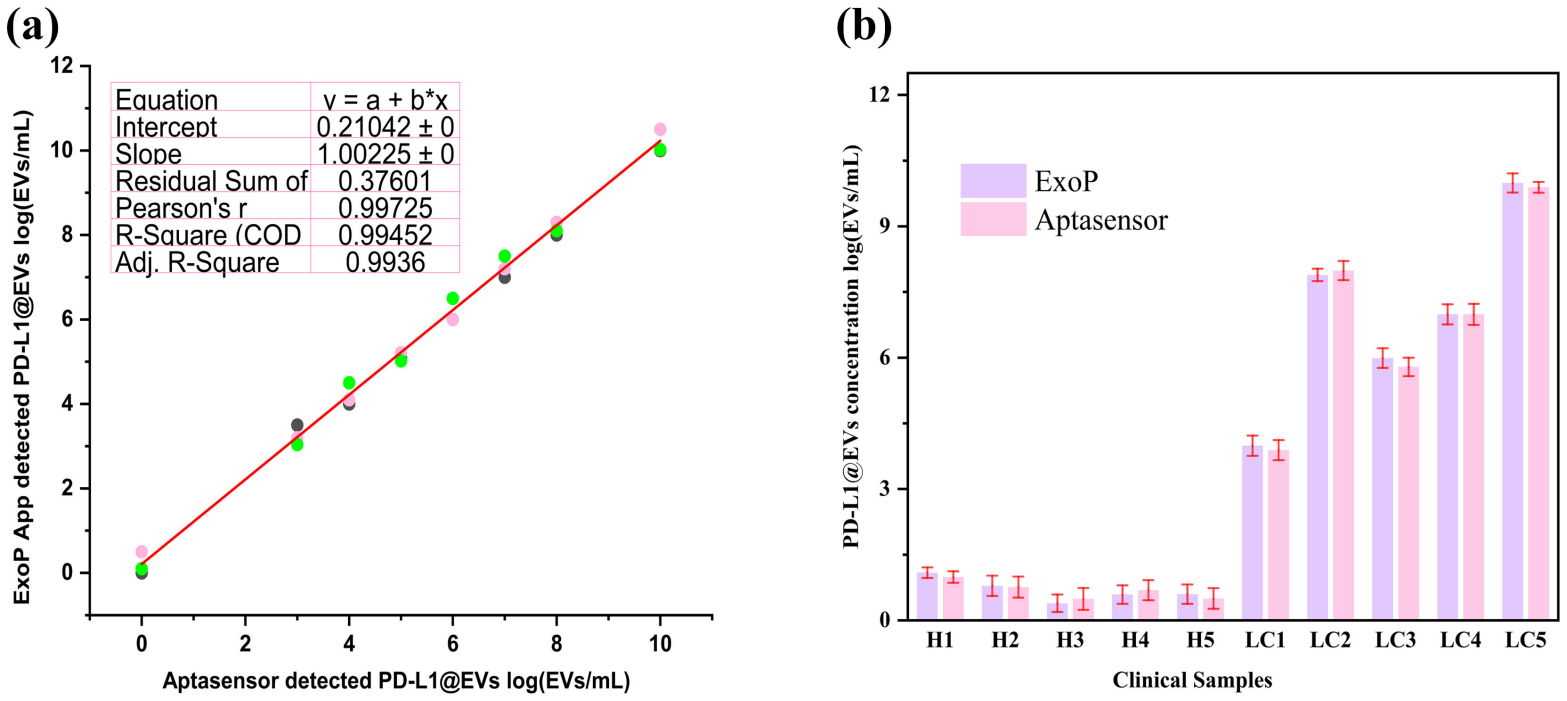
The ExoP smartphone app performance evaluations with colorimetric aptasensor spectroscopic readings. **(A)** The ExoP app performance along with the colorimetric aptasensor (absorbance at 400 nm) on the various concentrations of PD-L1@EVs. **(B)** ExoP app performance compared to aptasensor in quantifying PD-L1@EVs concentration from chromaticity.

**Figure 10A** shows that the intelligent ExoP detected a concentration of PD-L1@EVs that was quite close to the concentration detected using the aptasensor univariate analysis. Similarly, we also tested the ExoP performance using 10 clinical samples (5 healthy volunteers and 5 lung cancer patients). It can be seen in **Figure 10B** that there was no significant difference between the quantitative values of chromaticity revealed by the colorimetric aptasensor and the ExoP app for PD-L1@EVs.

## 4 Discussion

A huge chunk of research provides evidence that EVs are promising liquid biopsy markers for cancers, including lung cancer (19, 58). EVs are regarded as ideal biomarkers for their stability, non-invasive mode of acquisition, rich content, and recognition as a representative of the pathophysiological status of the originating cells (59). PD-L1@EVs have been recognized for their role in immunotherapy for lung cancer (60). Monitoring of PD-L1@EVs can also provide information about the response of tumors to treatment. PD-L1@EVs have been indicated as diagnostic and prognostic markers for lung cancer (61). In addition, conventionally, PD-L1 expression analysis is performed using the sample obtained through tissue biopsy, but it is not always feasible in the initial sample due to the insufficient quantity of tumor material and the difficulties in performing a new invasive biopsy. Therefore, PD-L1@EV analysis through liquid biopsy can be a valuable approach because it may better reflect tumor heterogeneity compared with the tissue biopsy and also due to its non-invasive nature (62).

Therefore, the simple and accurate detection of PD-L1@ EVs is crucial. To achieve the above objective, we developed a colorimetric aptasensor by integrating rapid capturing of EVs by (Fe_3_O_4_)-SiO_2_-TiO_2_ mag-nanoparticles with an HCR signal amplification strategy to detect PD-L1-positive EVs colorimetrically, achieving high sensitivity (LOD 3.6×10^2^ EVs/mL) and specificity. To further simplify the quantitative analysis of chromaticity, we trained and tested a CNN-based deep learning model and integrated it into a user-friendly smartphone app (named ExoP). Both the model and ExoP showed excellent accuracy in predicting the concentration of PD-L1@EVs by analyzing the chromaticity of the tubes. As reported by Tania (41), deep learning approaches integrated into smartphone apps can provide efficient tools that can be helpful in eliminating the subjectivity of chromaticity interpretation and can provide quantitative information without any additional instrument. Many of the strategies used for detecting EVs have involved a time-consuming ultracentrifugation process or the use of commercial kits to isolate EVs from clinical samples before detection, which can result in a lengthy process and an increased risk of contamination (21). Some of these methods have been validated using simulated serum or diluted serum with added EVs, or by detecting EVs in cell culture medium. However, the colorimetric aptasensor demonstrated highly accurate results when testing real, undiluted serum samples. The colorimetric aptasensor for PD-L1@EVs showed excellent performance: a wide linear range from 10^3^ to 10^8^ EVs/mL and an LOD as low as 3.6×10^2^ EVs/mL compared to the majority of previous methods, as tabulated in **Table 1**. Detection approaches involving immune-affinity-based capturing of EVs by membrane markers can be excellent in sensitivity and reproducibility, making them ideal for biomarker discovery. However, immune-affinity methods are not feasible for large-volume samples as biological ligands are costly. Another drawback is the heterogeneity of the EV population; for instance, a CD63 membrane marker is absent, but PD-L1 is present on the surface of EV, which can risk the missing of a specific population of EVs (70).

**Table 1:** Comparison with previous methods.

Method	LOD (EVs/mL)	Sample type	Prior isolation (UC/kit/UF)	Linear range (EVs/mL)	Ref.
Electrochemical	2.67 × 10^4^	Spiked serum	Yes	5 × 10^4^–5 × 10^9^	(63)
Electrochemical	1.5 × 10^6^	FBS	Yes	2 × 10^6^–4 × 10^8^	(64)
Fluorescence	7.6 × 10^6^	Culture medium	Yes	1.6 × 10^7^–4.2 × 10^10^	(65)
Colorimetric	13.5 × 10^8^	Diluted serum	Yes	1.9 × 10^9^–3.38 × 10^10^	(66)
Chemiluminescence	2.63 × 10^8^	Undiluted serum	No	2.9 × 10^8^–2.8 × 10^11^	(67)
Chemiluminescence	2.85 × 10^5^	Undiluted serum	No	10^5^−10^8^	(23)
Mass spectrometry	3.05 × 10^5^	Plasma	No	1 × 10^6^–5 × 10^7^	(68)
Impedance	1.4 × 10^4^	Serum	Yes	1.0 × 10^5^–1.0 × 10^9^	(69)
Colorimetric	3.6 × 10^2^	Serum	No	10^3^–10^8^	This work

Therefore, efficient, rapid capturing of EVs through the lipid bilayer by TiO_2_ is highly valuable (19, 22), and it can be commonly applied to a wide range of biological samples to accelerate the development of EV detection platforms with clinical application.

Colorimetric approaches are preferred for their simplicity and robust naked-eye qualitative detection. However, qualitative determination by the naked eye can lead to significantly biased readings and unreliable analyses. Therefore, many scientific groups have developed smartphone app-based image capturing and deep learning analysis for colorimetric approaches in the area of disease diagnosis (71). *The GlucoSensing* app was developed utilizing machine learning with a remote server for image processing and feature extraction to predict the level of glucose in unknown saliva samples (72). Another study revealed the use of a CNN-assisted app for the prediction of glucose levels in urine from images of colorimetric sensors using gold nanoparticles. In **Table 2**, we tabulated different articles using machine learning algorithms and smartphone images for various detection approaches and targets.

**Table 2:** Performance comparisons of our model with previous models following the same approach.

Target	Approach	Images	Model	Performance	Ref.
Urine glucose	Colorimetry	Smartphone	Faster-RCNN	*R* ^2^ = 0.97	(57)
Living cells	Colorimetry	Smartphone	Mask-CNN	Accuracy = 95%	(73)
Lactate	Colorimetry	Smartphone	CNN	Accuracy = 99%	(74)
Virus	Fluoresce	Smartphone	CNN	Accuracy = 98%	(75)
*E. coli*	Colorimetric	Smartphone	CNN	Accuracy = 97%	(76)
Bacterial biomarker	Raman spectroscopy	Spectra	CNN	*R* ^2^ = 0.97 MAE = 0.27	(77)
PCA3	Electrochemical	SEM images	SVM	Accuracy = 99%	(78)
PD-L1@EVs	Colorimetry	Smartphone	CNN	Accuracy = 99% AUROC = 0.99 MAE = 0.01 *R* ^2^ = 0.99	This work

SVM, Support vector machine; MAE, mean absolute error.

Our deep learning model has shown excellent output for various performance metrics, thereby validating its utility. We believe that the addition of the deep-learning-powered smartphone app has converted our colorimetric aptasensor into an intelligent colorimetric aptasensor capable of predicting the concentration of PD-L1@EVs based on the underlying decision system. Smartphone apps based on deep learning models pave the way for quantitative colorimetric detection with higher accuracy and repeatability (79). The potential of EVs for lung cancer diagnosis has been proven by a series of studies, and by combining the advantages of non-invasive EV-based detection with existing tests such as LDCT and other molecular markers, we can have the advantage of detecting and diagnosing lung cancer much earlier than through conventional, invasive, and risky tissue biopsy (3).

## 5 Conclusion

To summarize, a versatile colorimetric aptasensor for PD-L1@EVs was developed by harnessing (Fe_3_O_4_)-SiO_2_-TiO_2_ mag-nanoparticles to rapidly capture EVs from cell culture and patient serum. The specific PD-L1 marker on the EVs was recognized through an aptamer (PD-L1 aptamer) that provides an initiation sequence for HCR, resulting in long strands with many HRPs for signal amplification. HRPs catalyze the conversion of the substrate DA into PDA, resulting in a color change. The change in chromaticity corresponds to the concentration of PD-L1@EVs. The colorimetric aptasensor was able to detect PD-L1@EVs at concentrations as low as 3.6 × 10^2^ EVs/mL and a linear range of 10^3^–10^8^ EVs/mL. Colorimetric approaches still require instruments such as a spectrophotometer to quantify the results. To overcome this problem, a deep learning (CNN) model was trained and tested using the pre-obtained images for chromaticity analysis that can later automatically quantify the chromaticity of the new unseen images. The learned model was embedded into a custom-designed smartphone app named ExoP.

We envision that such an approach can provide a reliable alternative to traditional laboratory-based analytical instrumentation such as a spectrophotometer, which is generally a bulky and costly object and often requires a separate computer to function and skilled personnel to operate. No prior methodology was able to demonstrate an intelligent deep-learning-powered app such as ExoP for the quantification of chromaticity using images of the colorimetric aptasensor for EVs. The designed framework for chromaticity analysis with minimal user intervention or additional hardware attachments can be a useful intervention. We believe that EV-based non-invasive liquid biopsy approaches can be vital for the detection of lung cancer at earlier stages, which are amenable to treatment compared to traditional, highly invasive, and risky tissue biopsy.

## Data Availability

The original contributions presented in the study are included in the article/**Supplementary Material**. Further inquiries can be directed to the corresponding authors.
