# Supplementary material for: Colorimetric aptasensor coupled with a deep-learning-powered smartphone app for programmed death ligand-1 expressing extracellular vesicles
Source: Front Immunol. 2025 Jan 23;15:1479403. doi: 10.3389/fimmu.2024.1479403 (PMC11798968; doi:10.3389/fimmu.2024.1479403)
Supplement: Supplementary file 1 [file DataSheet1.docx]

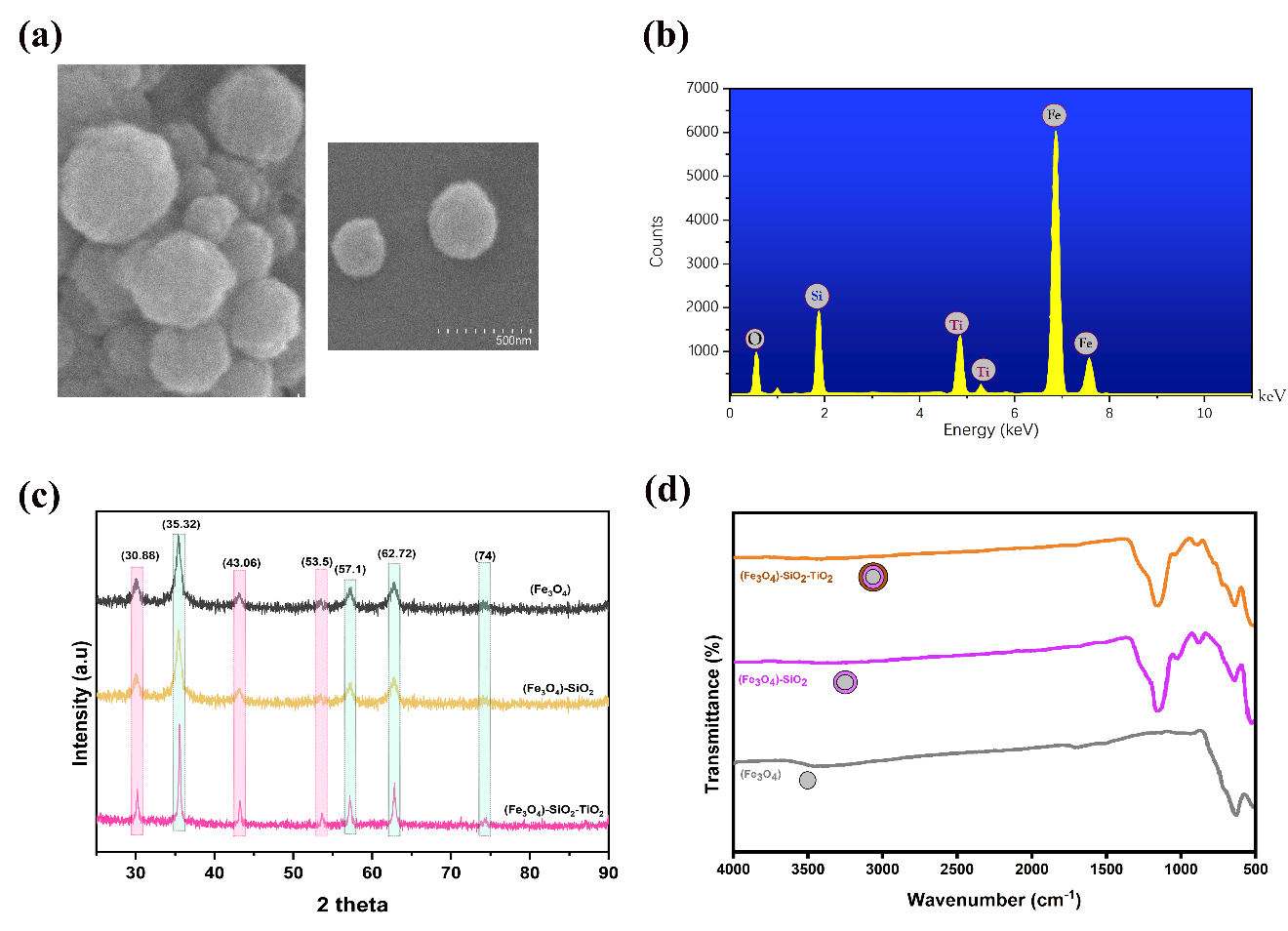


**Figure S1 .** Technical confirmations of (Fe_3_O_4_)-SiO_2_-TiO_2_ formation. (a) SEM images (b) EDS analysis (c) XRD analysis showing peaks at 2 theta 30.88,35.22,43.06,53.5, 57.1,62.72, and 74 represents cubic phase of Fe_3_O_4_ and anatase phase of TiO_2_ (JCPDS no. 21-1272) (d) FT-IR analysis showing peaks at 802 and 1068 cm^-1^ confirming silica coating, peak at 970 cm^-1^ confirm addition of titanium dioxide, and peaks in the range of 500–700 cm^-1^ can be attributed to Ti–O–Ti and Ti–O–Si.

**Table S 1** Information of all nucleotide sequences used in colorimetric aptasensor design.

| **Name** | **Sequence 5’-3’** |
| --- | --- |
| PD-L1 Aptamer | AGTCTAGGATTCGGCGTGGGTTAATTTTTTTTTACGCTCGGATGCCACTACAGACGGGCCACATCAACTCATTGATAGACAATGCGTCCACTGCCCGTCTCATGGACGTGCTGGTGAC |
| H1 | TTAACCCACGCCGAATCCTAGACTCAAAGTAGTCTAGGATTCGGCGTG |
| H2 | AGTCTAGGATTCGGCGTGGGTTAACACGCCGAATCCTAGACTACTTTG |
| Biotin -H1 | Biotin-TACCAGTGCGATGCTCAGTGCCGTATCTACACTTAGT  AGAAATTACCCTATAGTGAGTCGTATTAATTTC |
| Biotin-H2 | Biotin-TACCAGTGCGATGCTCAGTGCCGTTTCATCTACACT  TAGTAGAAATTACCCTATAGTGAGTCGTATTAATTTC |
| PD-L1-Biotin | AGTCTAGGATTCGGCGTGGGTTAATTTTTTTTTACGCTCGGATGCCACTACAGACGGGCCACATCAACTCATTGATAGACAATGCGTCCACTGCCCGTCTCATGGACGTGCTGGTGAC-biotin |

**Table S2** Configuration of the deep learning model.

| **Model configuration** | |
| --- | --- |
| Two-dimensional Convolution Pool Layer  (Extracts features from the input image) | Kernels=32, Kernel size=3x3, Stride=1. Pooling=max pooling (2x2) stride= 2, SELU activation |
| Two-dimensional Convolution Pool Layer  (Further extracts and refines features based on the previous layer's output) | Kernels=64, Kernel size=3x3, Stride=1. Pooling=max pooling (2x2) stride=2. |
| Two-dimensional Convolution Layer  (To extracts even higher-level features or combines existing features) | Kernels=128, Kernel size=5x5, Stride=1. Pooling=max pooling (2x2) stride= 2. |
| Fully Connected Layer  To flattened the output from the previous layer (all feature maps are converted into a single vector) and made the prediction for PD-L1@EVs concentration | Consisted of 64 neurons. |
| Output Layer  (Makes the final prediction for PD-L1@EVs concentration) | Consisted of one neuron |

Table S3: Details of the healthy samples (light green shade) and lung cancer patients (light yellow shade) used for clinical validation of the colorimetric aptasensor (green shade healthy and yellow shade patients)

| **S/No** | **Age** | **Sex** | **CEA**  **(ng/mL）** | **CA-125（U/mL）** | **CYFRA21-1**  **(ng/mL）** | **NSE**  **(ng/mL）** | **EGFR** | **T** | **N** | **M** | **Stage** |
| --- | --- | --- | --- | --- | --- | --- | --- | --- | --- | --- | --- |
| **1** | 70 | F | ____ | ____ | ____ | ____ | ____ | ____ | ____ | ____ | ____ |
| **2** | 60 | F | ____ | ____ | ____ | ____ | ____ | ____ | ____ | ____ | ____ |
| **3** | 73 | M | ____ | ____ | ____ | ____ | ____ | ____ | ____ | ____ | ____ |
| **4** | 30 | F | ____ | ____ | ____ | ____ | ____ | ____ | ____ | ____ | ____ |
| **5** | 42 | M | ____ | ____ | ____ | ____ | ____ | ____ | ____ | ____ | ____ |
| **6** | 29 | M | ____ | ____ | ____ | ____ | ____ | ____ | ____ | ____ | ____ |
| **7** | 75 | M | ____ | ____ | ____ | ____ | ____ | ____ | ____ | ____ | ____ |
| **8** | 50 | M | ____ | ____ | ____ | ____ | ____ | ____ | ____ | ____ | ____ |
| **9** | 65 | M | ____ | ____ | ____ | ____ | ____ | ____ | ____ | ____ | ____ |
| **10** | 43 | M | ____ | ____ | ____ | ____ | ____ | ____ | ____ | ____ | ____ |
| **11** | 93 | F | ____ | ____ | ____ | ____ | ____ | ____ | ____ | ____ | ____ |
| **12** | 42 | M | ____ | ____ | ____ | ____ | ____ | ____ | ____ | ____ | ____ |
| **13** | 69 | M | ____ | ____ | ____ | ____ | ____ | ____ | ____ | ____ | ____ |
| **14** | 75 | M | ____ | ____ | ____ | ____ | ____ | ____ | ____ | ____ | ____ |
| **15** | 70 | F | ____ | ____ | ____ | ____ | ____ | ____ | ____ | ____ | ____ |
| **16** | 26 | M | ____ | ____ | ____ | ____ | ____ | ____ | ____ | ____ | ____ |
| **17** | 37 | F | ____ | ____ | ____ | ____ | ____ | ____ | ____ | ____ | ____ |
| **18** | 49 | M | ____ | ____ | ____ | ____ | ____ | ____ | ____ | ____ | ____ |
| **19** | 47 | M | ____ | ____ | ____ | ____ | ____ | ____ | ____ | ____ | ____ |
| **20** | 49 | F | ____ | ____ | ____ | ____ | ____ | ____ | ____ | ____ | ____ |
| **21** | 30 | F | ____ | ____ | ____ | ____ | ____ | ____ | ____ | ____ | ____ |
| **22** | 69 | M | 2.54 | 27.0 | 5.4 | 21.0 | + | T2b | N2 | M0 | IIIA |
| **23** | 50 | M | 3.07 | 23.3 | 7.3 | 11.5 | + | T1a | N2b | M0 | IIIA |
| **24** | 87 | F | 4.07 | 35.1 | 3.2 | 15.2 | + | T1b | N2a | M0 | IIB |
| **25** | 82 | M | 2.2 | 16.98 | 2.10 | 12.15 | + | T2b | N0 | M0 | IIA |
| **26** | 46 | M | 2.00 | 13.9 | 2.00 | 13.00 | + | T2a | N1 | M1a | IVA |
| **27** | 94 | F | 9.00 | 18.7 | 2.64 | 15.9 | + | T3 | N1 | M0 | IIIA |
| **28** | 34 | M | 3.99 | 7.00 | 2.15 | 14.00 | + | T1a | N1 | M0 | IIA |
| **29** | 58 | M | 1.08 | 25.1 | 4.32 | 10.00 | + | T2a | N0 | M0 | IB |
| **30** | 61 | F | 30.10 | 47.00 | 11.01 | 21.00 | + | T3 | N0 | M0 | IIB |
| **31** | 49 | M | 19.97 | 42.13 | 9.12 | 15.09 | + | T2b | N2a | M0 | IIIB |
| **32** | 95 | F | 14.4 | 6.8 | 14.00 | 29.10 | + | T3 | N3 | M1a | IVA |
| **33** | 57 | M | 17.00 | 32.1 | 13.12 | 19.18 | + | T1c | N3 | M0 | IIIB |
| **34** | 46 | M | 40.81 | 69.04 | 19.99 | 37.90 | + | T1a | N2b | M0 | IIIA |
| **35** | 81 | F | 33.08 | 79.5 | 12.81 | 22.81 | + | T1c | N3 | M1b | IVA |
| **36** | 73 | M | 98.00 | 59 | 5.00 | 15.93 | + | T4 | N3 | M0 | IIIC |
| **37** | 53 | M | 12.20 | 19.81 | 4.10 | 15.10 | + | T2b | N2a | M1c | IVB |
| **38** | 63 | F | 20.00 | 10.00 | 12.00 | 11.10 | + | T4 | N2b | M1b | IVA |
| **39** | 61 | M | 19.10 | 17.40 | 12.14 | 11.91 | + | T1c | N2b | M1a | IVA |
| **40** | 72 | M | 13.19 | 17.30 | 4.05 | 20.00 | + | T2a | N2b | M0 | IIIB |
| **41** | 76 | M | 11.48 | 35.1 | 14.12 | 11.50 | + | T3 | N3 | M1c | IVB |
| **42** | 57 | M | 30.10 | 27.00 | 15.01 | 20.40 | + | T4 | N2b | M1a | IVA |
